# Association between genetic taste sensitivity, 2D:4D ratio, dental caries prevalence, and salivary flow rate in 6-14-year-old children: a cross-sectional study

**DOI:** 10.15171/joddd.2016.023

**Published:** 2016-08-17

**Authors:** Chintamaneni Raja Lakshmi, Doppalapudi Radhika, Mpv Prabhat, Sujana mulk Bhavana, Nallamilli Sai Madhavi

**Affiliations:** ^1^Reader, Department of Oral Medicine and Radiology, Drs Sudha and Nageswara Rao Siddhartha Institute of Dental Sciences, Chinnaoutpalli, Gannavaram Mandal-521286, India; ^2^Postgraduate Student, Department of Oral Medicine and Radiology, Drs Sudha and Nageswara Rao Siddhartha Institute of Dental Sciences, Chinnaoutpalli, Gannavaram Mandal-521286, India; ^3^Professor and Head of the Department, Department of Oral Medicine and Radiology, Drs Sudha and Nageswara Rao Siddhartha Institute of Dental Sciences, Chinnaoutpalli, Gannavaram Mandal-521286, India; ^4^Assistant Professor, Department of Oral Medicine and Radiology, Drs Sudha and Nageswara Rao Siddhartha Institute of Dental Sciences, Chinnaoutpalli, Gannavaram Mandal-521286, India

**Keywords:** Dental caries risk, genetic taste sensitivity, propylthiouracil, saliva

## Abstract

***Background.*** The aim of this study was to assess the relationship between genetic taste sensitivity, dietary preferences and salivary flow rate in 6‒14-year-old children for identification of individuals at higher risk of developing dental caries.

***Methods.*** A total of 500 children 6‒14 years of age, of both genders, who reported to the Department of Oral Medicine and Radiology, were included. Propylthiouracil (PROP) sensitivity test was carried out and the subjects whose perception was bitter were grouped as tasters, whereas those who were unable to perceive any taste were grouped as non-tasters. The 2D:4D ratio was obtained by measuring the length ratio of index finger to ring finger with the help of a digital Vernier caliper. Evaluation of dietary preferences was carried out using a 24-hour dietary recall and accordingly they were categorized as sweet likers and dislikers. The salivary flow rate was estimated by collecting unstimulated saliva by spitting method. Data were analyzed with Student’s t-test and chi-squared test.

***Results.*** The results suggested a positive relation between low digit ratio (2D:4D), non-tasters, sweet likers and high caries index among the participants with a highly significant statistical difference (P ≤ 0.000). Tasters had high mean of USSR (0.48) than non-tasters (0.29), which was statistically significant.

***Conclusion.*** The present research revealed a positive correlation between all the parameters evaluated. Therefore an individual considered as non-taster by PROP was a sweet liker with low 2D:4D ratio, reduced salivary flow rate and high caries index.

## Introduction

Dental caries is the worldwide infectious disease of the oral cavity.^1^ It is most common among children. Unlike other problems, it is irreversible in nature and cannot be controlled by pharmacological interventions and may lead to pain, with harmful effects on the oral cavity, hindering the nutritional status and growth of a child.^2^ The positive correlation between high sugar intake and the caries risk has been documented in the literature; however, recent concepts like genetic taste sensitivity and taste thresholds have been evolving to identify the caries risk at an early stage.^3^

Genetic taste sensitivity dictates the fondness or refusal of food by children, who are accordingly categorized into tasters or non-tasters. PROP is the drug used for the treatment of Graves disease. In clinical practice^the^bitter taste of PROP was found to be a consistent factor for assessing genetic taste sensitivity levels, which in turn appeared to be influenced by TAS2R38 gene.^4^

Therefore, evaluation of individuals’ taste threshold using PROP will be helpful for identifying children at high caries risk. In addition, the current research enquires the role of 2D:4D ratio in taste behavior. The ratio is said to be sexually differentiated in humans and the probable mechanism is that the common genes Hox A and Hox D underlie the development of both digits and gonads, which in turn are known to influence the taste behavior in the human brain.^5^Hence this biological marker can also be used to predict children’s caries susceptibility by determining their dietary preferences, taste perception and also the association between them.^6^ In addition, sialometry has gained popularity in recent years for assessing the caries risk due to its non-invasive nature. With this background, the aim of the present study was to assess the association between genetic taste sensitivity using PROP, 2D:4D ratio, salivary flow rate and the dental caries prevalence in 6‒14-year-old children, to identify caries prone individuals, so as to enable a preventive program for them.

## Methods

All the procedures in this study were in compliance with the Helsinki Declaration, and the ethical clearance was obtained from the concerned committee for this research protocol.

### Participants

A pilot study was carried out to assess the feasibility of the study, and sample size calculations were carried out relying on the results gained in the study. A total sample of 450 was sufficient to detect a statistically significant difference of 10% with 95% confidence interval and considering the design effect of two. Assuming 10% loss of data due to rejections, errors, etc, the sample size increased to 500.

Five hundred children, 6‒14 years of age, of both genders, reporting to the Department of Oral Medicine and Radiology, were selected by systematic random sampling method based on inclusion and exclusion criteria.^5-7,8^

### Inclusion criteria

- An age range of 6‒14 years.
- Individuals with stable mental status.
- Subjects not under any prescription influencing the rate of salivary flow.
- Subjects with no known previous history of allergic reactions to 6-n-propylthiouracil.
- Subjects not under specific dental caries preventive program.

### Exclusion criteria

- Children without parents’ consent.
- Subjects taking antidepressants and antibiotics.
- Children who cannot be included under class I American Society of Anesthesiologists (ASA) physical status.
- Subjects under orthodontic therapy.

### Preparation of the strips

**Figure 1.**
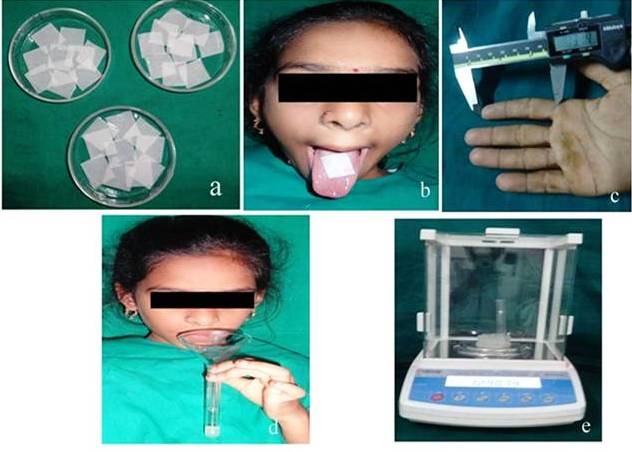
Whatmann filter papers dissolved in PROP solution (a). PROP strip placed on the dorsum of the tongue (b). Measuring the index digit length using digital Vernier callipers (c). Collection of saliva by spitting method (d). Weighing the device used for measurement of saliva (e).

Uncontaminated PROP samples were procured from the pharmaceutical company (Swapnroop, Mumbai, India) and the PROP strips were prepared at Pharmaceutical Institute. Whattman filter paper was prepared in the required size of 2×2 cm and sterilized in an autoclave at 121°C for 15 minutes. These strips were pre-weighed and stored in desiccators. 10 mL of ethyl alcohol was used to dissolve 10 mg/mL of PROP in a beaker.^9^ Ten autoclaved Whattman filter paper strips were soaked for a period of one hour to allow absolute absorption of PROP (Figure 1a). These soaked strips were taken out of beaker and kept for drying at room temperature. The difference between the pre-weighed and the post-weighed strips provided the appropriate amount of drug infused on each strip, which was calculated at 1.6 mg of PROP for each strip.^4^

### PROP sensitivity test

PROP sensitivity test was performed on the children to evaluate the genetic sensitivity levels of bitter or sweet substances. PROP sensitivity test was carried out by keeping a prepared strip for 30 seconds on the dorsal two-thirds of the tongue (Figure 1b). It was ensured that the subjects refrained from consuming any diet for a period of two hours prior to PROP testing and they were advised to thoroughly clean their mouth with distilled water prior to the test. These children were categorized into two groups as PROP tasters and PROP non-tasters based on their ability of taste perception.^4^ Those who tasted bitter were dubbed tasters and those unable to perceive any taste were categorized as non-tasters.

### 2D:4D Ratio

The ratio of the lengths of the index finger (2D) and ring finger (4D) was determined using digital Vernier calipers (Figure 1c) and the study participants were categorized into high 2D:4D, low 2D:4D and equal 2D:4D digit ratio.^4^

### Dietary preferences

Evaluation of dietary preferences was carried out using a 24-hour dietary recall. The subjects were asked to report the item, quantity of food usually consumed, along with frequency over a definite period of time, i.e. at specific time intervals. The data collected was again reconfirmed with the subjects’ parents who were accompanying them at that particular time. The type of sugar consumed was considered as liquid, solid, sticky and slowly dissolving along with their frequencies. A sweet score of 5 or less was considered as excellent, with a score of 10 as good and a score of 15 or more as watch-out zone.^11^Based on this data, the subjects in the watch-out zone were grouped into sweet likers and the subjects in the two other groups were considered as sweet dislikers.

### Dental caries experience

The caries experience (DMFT/dft index) was taken and subjects with a total DMFT/dft score of >5 were considered to have a high caries rate.^12,13^

### Collection of unstimulated saliva

Saliva was collected under standard temperature and humidity between 9:30 am to12:30 pm in order to avoid the effects of circadian rhythm.^14^ The participants were advised not to eat, drink and smoke one and a half hour prior to the sample collection. Children were asked to rest for five minutes and to gulp down all salivary remnants before sample collection. They were advised to bend the chin forward to pool the saliva in the floor of the mouth and let saliva flow into a test tube through a funnel (Figure 1d). The whole salivary sample which was collected for a period of 5 minutes was then calculated. The ratio of the difference between the pre-weighed and post-weighed test tubes to the duration of collection of saliva was expressed as mL/min (Figure 1e).^15^ Clinical examinations for dental caries experience, unstimulated saliva measurements and PROP sensitivity test were carried out by a single investigator.

### Statistical analysis

Data were analyzed with SPSS (Version 20, Chicago Inc.), using Pearson’s correlation coefficient, chi-squared test and Student’s t-test. Statistical significance was set at P ≤ 0.05.

## Results

**Figure 2.**
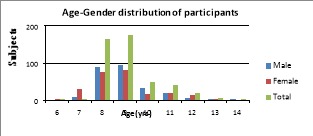
Age-gender distribution.

**Figure 3.**
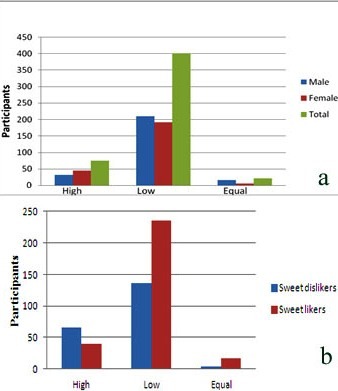
Distribution of study participants based on digit ratio (a). Distribution of dietary preferences in terms of digit ratio (b).

Out of 500 children selected for study, two were excluded due to incomplete data. Among the 498 participants, 176(35.3%) of them were 9-year old, 164(32.9%) were 8-year old school children and almost equal gender wise distribution (Figure 2). When 2D:4D ratio was measured with Vernier calipers, majority (81.4%) had low 2D:4D ratio, only 15% of them had high 2D:4D ratio and very few (4.6%) had equal digit ratio. Among the males, 81.7% of them had low 2D:4D ratio, 12% had high 2D:4D ratio and remaining males had equal 2D:4D ratio; 79.6% of the females had low 2D:4D ratio, 18.2% had high 2D:4D ratio and 1.2% females had equal 2D:4D ratio ( (Figure 3a) Comparison between PROP sensitivity and gender was shown in Table 1, 140 out of 257 males and 111 out of 241 females were non tasters of PROP and the remaining were considered as tasters. Majority of the non-tasters in both the genders had low 2D:4D ratio which was statistically significant (P = 0.000).

**Table 1:** Distribution of tasters and non-tasters in terms of gender and digit ratio

**Parameter**	**High 2D:4D ratio**	**Low 2D:4D ratio**	**equal 2D:4D ratio**	**Total**	**Chi square**	
					**Test value**	**P value**
**Male**	**31** 16(NT)+15(T)	**210** 115(NT)+59(T)	**16** 9(NT)+7(T)	257	1209.69	0.000
**Female**	**44** 21(NT)+23(T)	**192** 86(NT)+106(T)	**05** 4(NT)+2(T)	241		
**Total**	75(15%)	402(80.7%)	21(4.3%)	498(100)		

Dietary preferences were assessed from 24-hr dietary recall history, on a whole 59% of the participants were sweet likers and 189 out of 293 sweet likers were non tasters for PROP sensitivity which was statistically significant(P ≤ 0.000; Table 2). When dietary preferences were compared with digit ratio, 80% of the sweet likers had low 2D:4D ratio and only 66.6% of the sweet dislikers had low 2D:4D ratio, 31.7% of sweet dislikers and 13.5% of sweet likers had high 2D:4D ratio which was statistically significant (P ≤ 0.000; Figure 3b).

**Table 2:** Distribution of dietary preferences and tasters-nontasters

**Parameter**	**Tasters**	**Non-tasters**	**Total**	**Chi square**	
				**Test value**	**P value**
**Sweet dislikers**	109	96	205(41%)	22.492	0.000
**Sweet likers**	104	189	293(59%)		
**Total**	217	281	498		

Non tasters have more Dental caries experience with mean DMFT value 1.7±0.4 where as for tasters it was 1.1 ± 0.8 with statistically significant p value (Table 3). The mean score for the rate of unstimulated saliva in tasters was 0.48±0.03 and for non tasters it was 0.29 ± 0.04 which was statistically significant (P ≤ 0.000; Table 3).

**Table 3:** Comparison between mean DMFT, USFR and PROP sensitivity

**Parameter**	**Taster**	**Non-taster**	**Student's t test**	
			**Test value**	**P value**
**Mean DMFT**	1.1±.8	1.7±.4	4.292	0.0123
**Mean USFR**	0.48±0.03	0.29±0.04	14.231	0.000

DMFT: Decayed, Missing, Filled Teeth; USFR: Unstimulated Salivary Flow Rate; PROP sensitivity: Propylthiouracil sensitivity.

## Discussion

PROP has been documented in the literature in relation to the genetic sensitivity to expect the response of individuals through hedonic scale to sweet taste in both males and females.^16,17^ Taste perception of humans varies according to their degree of taste sensitivity to phenylthiocarbamide (PTC) and its structurally similar drug referred to as PROP. Sensitivity of PROP strip is known to be a reliable test in assessing this genetic sensitivity to bitter taste, which is an inherent genetic feature.^16^

In the present study non-tasters and tasters of PROP were 57.4% and 42.6%, respectively, consistent with studies by Verma et al^5^ Pidamale et al,^4^ and contradicting with the results reported by Rupesh and Nayak,^18^ where the non-tasters were found to be 19.5%. In our study, the were more numerous female tasters (54.3%) compared to the males (45%), consistent with the findings of Rupesh and Nayak,^18^ which was attributed to the fact that increased number of fungiform papillae enhance the sensitivity to bitter taste as reported by Shahbake et al^19^ and Delwiche et al.^20^ Among the dietary preferences, the majority of the subjects who were dubbed as non-tasters were interpreted as sweet likers and studies also suggest that there are difference in food preferences between tasters and non-tasters, with Pidamale et al^4^ and Verma et al^9^ reporting that non tasters were sweet likers.

The 2D:4D ratio has been projected as a putative marker for prenatal hormone exposure as well as homeobox (HOX) and androgen receptor gene expression.^13^ The individual variability of 2D:4D is established in utero during the second trimester and appears stable during postnatal life. This evidence proposed that 2D:4D can be used as a useful indicator for assessing prenatal sex hormone action in the body, brain and behavior along with the taste perception.

In the present study, 81.7% of males and 79.6% of the females had low 2D:4D ratio, consistent with a study by Verma et al,^5^ who reported that 80% of the sweet likers had low 2D:4D ratio.

The dental caries experience in non-tasters was slightly higher compared to tasters (1.7 vs 1.1), which is similar to other studies by Verma et al^9^ and Rupesh and Nayak.^18^ These results can be attributed to the fact that most of the non-tasters were sweet likers. However, the overall mean DMFT of the study participants was in par with national mean DMFT of the same age group and the study area is known for endemicity of mild dental fluorosis, and fluoride might be considered a possible reason for increased sugar threshold in sweet likers compared to the study conducted by Verma et al,^9^ where mean DMFT in sweet likers was 5.4.

Pidamale et al^21^ and Wang et al^22^ reported that unstimulated flow rate has been proved to be a better indicator for salivary function rather than stimulated flow rate since it is mildly affected by systemic conditions. Therefore, unstimulated salivary flow rate was used for the first time in comparison to PROP sensitivity in our study and mean USSR in tasters was higher compared to non-tasters.

There were many risk indicators for dental caries as reported in the literature, of which diet is one of the modifiable risk factors and it is the common risk factor for many disorders such as obesity, diabetes mellitus, etc.^23^ Therefore, early detection of this common risk factor (preferring sweets) can help in planning preventive programs for such populations. PROP sensitivity test can be a valuable tool in the future to assess the inherent genetic sensitivity of a person for dietary preferences and it is a simple and cost-effective method compared to other caries risk assessment tools like cariogram. One of the limitations of this test was the fact that interventions based on PROP sensitivity might be valid only in children because studies have shown that in contrast to children, adults’ sweet predilection was not affected by genotyping of bitter receptors.^24,25^ Therefore, longitudinal studies were required in future to assess the validity of PROP sensitivity as a caries risk factor.

## Conclusion

The present research revealed a positive correlation between all the parameters evaluated. Therefore, individuals who were considered as non-tasters by PROP were sweet likers with low 2D:4D ratio, reduced salivary flow rate and high caries index. Thus the present study can be performed as a simple, non-invasive chair-side procedure for early detection of individuals with high caries risk.

## Acknowledgments

We would like to acknowledge Dr. Sudhakar, Professor in the Department of Public Health Dentistry, for his support and encouragement.

## Authors’ Contribution

The basic concept of the study, major drafting of the article, statistical analysis, and interpretation of data were carried out by CRL. DR carried all the clinical procedures and PROP sensitivity tests. SMB performed salivary analysis as well as the refinement of the article. MPV was responsible for conducting the proper clinical procedures. NSM was responsible for the intellectual content and critical assessment of the article. All the contributors read the final proof of the article and approved its final version.

## Funding

The study was self-funded.

## Competing interests

The authors declare no competing interests with regards to authorship and/or publication of this article.

## Ethics approval

All the procedures followed in this study were in compliance with the Helsinki Declaration and the ethical clearance was obtained from the concerned committee for this research protocol.
